# Natural History of Arrhythmogenic Cardiomyopathy

**DOI:** 10.3390/jcm9030878

**Published:** 2020-03-23

**Authors:** Giulia Mattesi, Alessandro Zorzi, Domenico Corrado, Alberto Cipriani

**Affiliations:** Department of Cardiac, Thoracic, Vascular Sciences and Public Health, University of Padua Medical School, 35128 Padua, Italy; g.mattesi17@gmail.com (G.M.); alessandro.zorzi@unipd.it (A.Z.); alberto.cipriani@unipd.it (A.C.)

**Keywords:** sudden cardiac death, arrhythmogenic cardiomyopathy

## Abstract

Arrhythmogenic cardiomyopathy (AC) is a heart muscle disease characterized by a scarred ventricular myocardium with a distinctive propensity to ventricular arrhythmias (VAs) and sudden cardiac death, especially in young athletes. Arrhythmogenic right ventricular cardiomyopathy (ARVC) represents the best characterized variant of AC, with a peculiar genetic background, established diagnostic criteria and management guidelines; however, the identification of nongenetic causes of the disease, combined with the common demonstration of biventricular and left-dominant forms, has led to coin the term of “arrhythmogenic cardiomyopathy”, to better define the broad spectrum of the disease phenotypic expressions. The genetic basis of AC are pathogenic mutations in genes encoding the cardiac desmosomes, but also non-desmosomal and nongenetic variants were reported in patients with AC, some of which showing overlapping phenotypes with other non-ischemic diseases. The natural history of AC is characterized by VAs and progressive deterioration of cardiac performance. Different phases of the disease are recognized, each characterized by pathological and clinical features. Arrhythmic manifestations are age-related: Ventricular fibrillation and SCD are more frequent in young people, while sustained ventricular tachycardia is more common in the elderly, depending on the different nature of the myocardial lesions. This review aims to address the genetic basis, the clinical course and the phenotypic variants of AC.

## 1. Introduction

Arrhythmogenic cardiomyopathy (AC) is a heart muscle disease characterized by loss of ventricular myocardium and its replacement by fibrofatty tissue and the occurrence of ventricular arrhythmias (VAs), not explained by ischemic, hypertensive or valvular disorders [1]. Arrhythmogenic right ventricular cardiomyopathy (ARVC), an inherited heart muscle disease characterized histopathologically as fibrofatty infiltration of the right ventricular (RV) myocardium, represents the best characterized variant of AC, with early clinico-pathological descriptions [2,3], a peculiar genetic background [4], established diagnostic criteria [5] and management guidelines [6]; however, the identification of nongenetic causes of the disease, combined with the common demonstration of biventricular and left-dominant forms, has led to coin the term of “arrhythmogenic cardiomyopathy”, to better define the broad spectrum of the disease phenotypic expressions [1,7]. 

The genetic basis of AC consists of one of more pathogenic mutations in genes encoding the cardiac desmosomes, which are specialized structures in cell-cell adhesion. However, genotype-phenotype studies allowed to discover non-desmosomal variants in individuals fulfilling current diagnostic criteria for AC, some of which showing phenotypes in overlap with other cardiomyopathies, particularly dilated cardiomyopathy (DCM). Nongenetic disorders like myocarditis and sarcoidosis, are known to mimic AC, because of the similarity of the fibrotic lesions and the susceptibility to VAs. 

AC affects approximately one in 2000–5000 individuals and is one of the major causes of sudden cardiac death (SCD) in the young and athletes [4]. AC patients have a structurally normal heart at birth and develop the phenotype later on. In the first stages of the disease, nonspecific symptoms, like palpitations, chest pain and syncope, may occur without overt structural changes. Electrocardiographic (ECG) abnormalities typically precede the morpho-functional abnormalities, like ventricular dilatation, systolic dysfunction and regional wall-motion anomalies, which become more evident with the progression of the disease. The current treatment strategies focus on lifestyle intervention (restriction of sport activity), controlling symptoms and arrhythmias with anti-arrhythmic medications and catheter ablation, and preventing SCD with implantable cardioverter defibrillator (ICD) implantation. New targeted therapies are expected, based on advances in molecular genetics and biology and better understanding of complex molecular mechanisms underlying AC.

This review aims to summarize the available information regarding the genetic background, the clinical course and the phenotypic variants of AC.

## 2. Genetic Basis of AC

**Figure 1:**
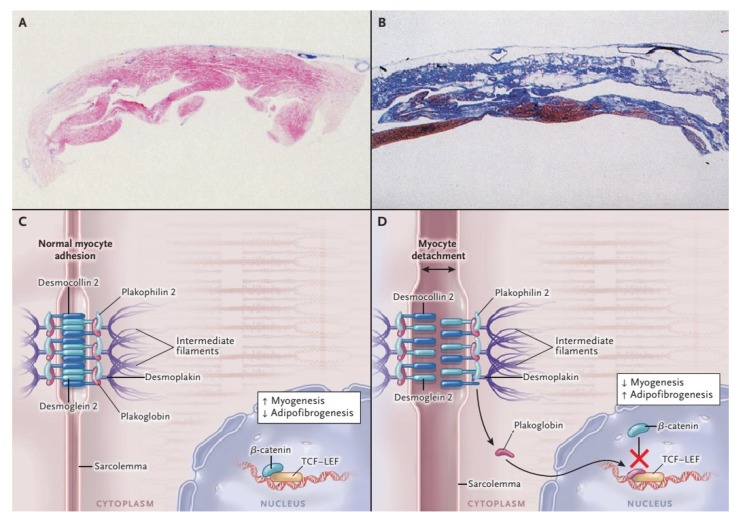
Histopathological Features and Pathogenesis of arrhythmogenic right ventricular cardiomyopathy (ARVC). With the azan trichrome stain, myocytes appear red, fibrous tissue appears blue, and fatty tissue appears white. Panel **A** shows a full-thickness histologic section (azan trichrome stain) of the anterior right ventricular wall in a normal heart; Panel **B** illustrates an analogous section from the heart of a patient with ARVC who died suddenly: Fibro-fatty tissue has replaced the muscular one. Desmosomes are not only structures supplying cell-cell adhesion, but they are also part of the Wnt–β-catenin signaling pathway, which suppresses the expression of adipogenic and fibrogenic genes (Panel **C** and **D**). Therefore, on one hand the impairment of desmosomal lead to detachment of cardiomyocytes (double-headed arrow), on the other in a gene transcriptional switch from myogenesis to adipogenesis and fibrogenesis (Panel **C** and **D**) [17]. Modified from Ref [4] with permission of the publisher.

**Figure 2:**
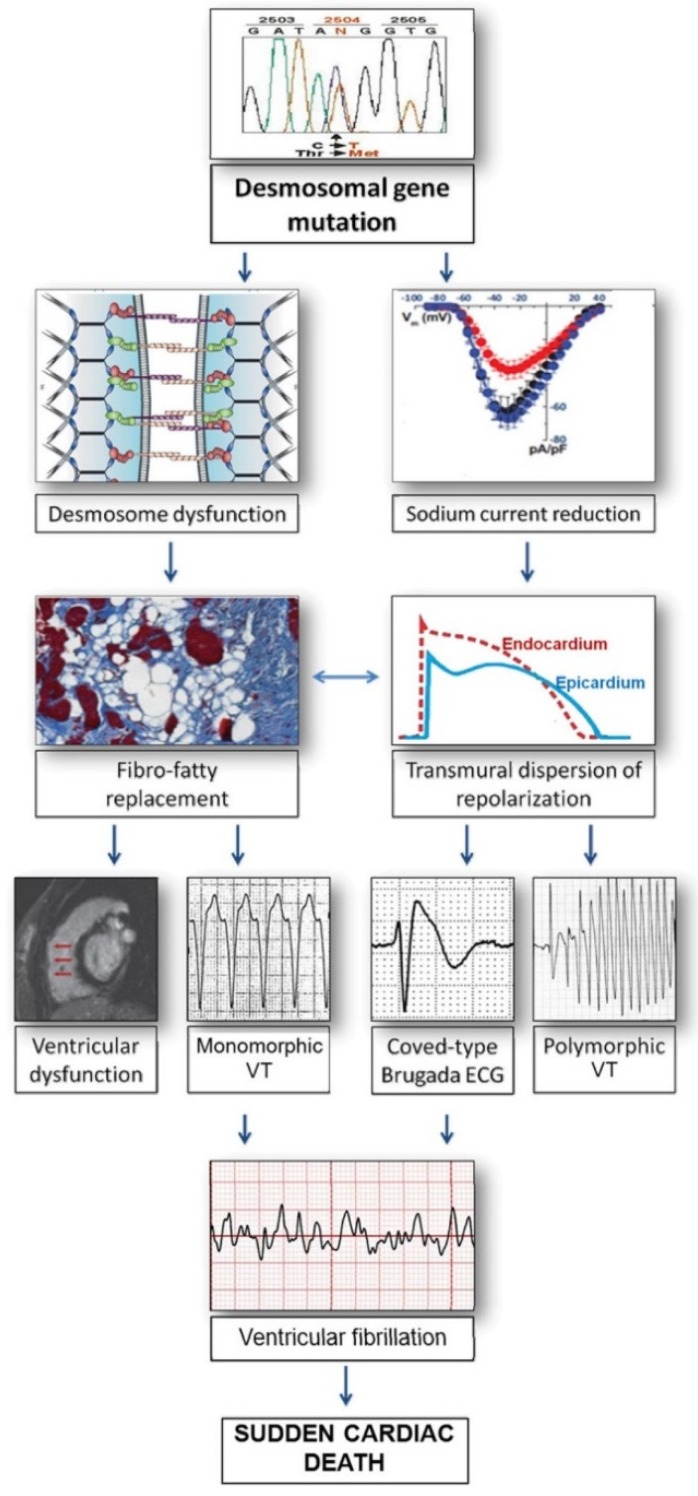
Relationship between arrhythmogenic right ventricular cardiomyopathy (ARVC) and Brugada Syndrome. Mutant desmosomal proteins may induce potentially lethal ventricular arrhythmias by causing gap-junction remodeling and modifying the amplitude and kinetics of the sodium current, as a consequence of the cross-talk between these molecules at the intercalated discs. According to this view, Brugada syndrome and ARVC may share clinical features and arrhythmic mechanisms because of their common origin from the connexome, a coordinated network of proteins involving desmosomes, sodium channels, and gap-junction, aimed to control synergistically adhesion, excitability, and coupling of myocardial cells [18,19,20]. ECG = electrocardiogram; VT = ventricular tachycardia. Modified from Ref [20] with permission of the publisher.

The familiar nature of AC was inspired by the first descriptions of two rare cardio-cutaneous syndromes named Naxos and Carvajal disease, characterized by palmoplantar keratosis, woolly hair and arrhythmic cardiomyopathy, inherited as an autosomal recessive trait [8,9]. The identification of homozygous mutations in the gene encoding plakoglobin (*JUP*) and desmoplakin (*DSP*) laid the foundations for the subsequent discovery of other desmosomal genes associated with AC, such as plakophilin2 (*PKP2*), desmoglein2 (*DSG2*)*,* and desmocollin2 (*DSC2*) [10,11,12,13,14]. Desmosomes are proteins which, together with the adherens junctions, gap junctions and ion channels, form the area composita at the intercalated disc. Since this structure is crucial for the electromechanical connection of cardiomyocytes and intracellular signaling cascades, mutant and impaired desmosomes lead to a decreased mechanical coupling between cardiomyocytes, favoring their detachment and subsequent cell death, inflammation and scarring [15]. The abnormal electric coupling and altered signal pathways due to remodeling at the intercalated discs are also implicated in the pathogenesis and arrhythmogenesis of AC [16], a detailed description of which is beyond the scope of this article. In Figure 1 and Figure 2 are briefly reported two molecular links between abnormal desmosomes, adipo-fibrogenesis and VAs predisposition [17,18,19,20]. 

Genes encoding non-desmosomal proteins like ion channels and cytoskeletal components have been also associated with phenotypes within the spectrum of AC, and this may confirm the “final common pathway” hypothesis, by which inherited cardiac diseases with similar phenotype and genetic heterogeneity are due to variants in genes encoding proteins of similar function or involved in a common pathway. According to this view, AC should be considered a disease not only of desmosomes, but of the intercalated disc as a whole [1]. In fact, mutations in transforming grow factor-3 (*TFGB3*) and in transmembrane protein 43 (*TMEM43*), which disrupt the desmosomal function, have been detected in patients with classical ARVC [21,22,23], as well as variants in αT-catenin (*CTTNA3*) [24]. Mutations in cadherin-2 (*CDH2*), a calcium-dependent cell surface adhesion molecule, have been found in families in which all the affected members presented VAs and AC [25,26]. Other examples of extra-desmosomal gene mutations which have been associated to AC are lamin A/C (*LMNA*), desmin (*DES*), filamin C (*FLNC*), and phospholamban (*PLN*), commonly presenting with a peculiar LV phenotype, characterized by a malignant arrhythmogenic predisposition represented by LV non-ischemic fibrosis [27,28,29,30,31,32,33]. In particular, truncating mutations in *FLNC* were associated with a LV phenotype including LV dilatation, systolic dysfunction and a subepicardial ring-like late gadolinium enhancement (LGE) pattern at cardiac magnetic resonance (CMR) imaging [30]. These patients were more prone to present frequent VAs and SCD [31]. Similarly, *DES*-p.Glu401Asp mutation carriers reported a high incidence of SCD [28], and showed extensive LV subepicardial circumferential fibrosis [29]. A distinct pattern of fibrosis was also identified in patients with *PLN*-p.Arg14del variants, who showed fibrofatty replacement predominantly at the LV posterolateral wall, associated with ECG low QRS voltages and malignant VAs and SCD [32,33]. 

Inheritance of most gene mutations associated with AC follows an autosomal dominant pattern with age-related, incomplete penetrance and variable expressivity. Unfortunately, Unfortunately the genetic cause remains to date undetermined in about 50% of probands with routine genetic testing [34]. Negative results could depend on many factors, like proband selection criteria, race, and ethnicity influence, unknown AC genes, and laboratory standard operating procedures, not including the evaluation of copy number variations of desmosomal genes, which recently showed the potential to increase the diagnostic yield of genetic testing [35]. Also, the detection of compound/digenic heterozygosity can be helpful, being identified in up to 25% of patients and accounting for both phenotypic variability and more malignant life-time arrhythmic outcome (dose–effect) [36,37,38]. On the other hand, several studies demonstrated the low specificity of the AC-associated genetic variants, finding them both in healthy individuals and in other cardiomyopathies [39,40]. This raised the need to reclassify many of them in variants of uncertain significance (an updated AC genetic database is accessible on www.arvcdatabase.info). 

The low sensitivity and the low specificity of molecular genetics recently raised concerns about its role in the diagnosis of AC. Unlike other cardiomyopathy, the 2010 International Task Force Criteria include the demonstration of a pathogenic variant in AC-related genes as a major criterion to establish the diagnosis. To date, it appears still challenging both to consider a gene mutation as disease-causative and to confer it pathogenicity. Accordingly, genetic testing should be reserved to probands who already have a “phenotypic” diagnosis of AC, and to family members [7].

## 3. Clinical Course

Natural history of AC depends on both cardiac electrical instability and progressive deterioration of cardiac performance. The disease can express with different variants embracing a large spectrum of phenotypic expressions, ranging from asymptomatic individuals without morphological abnormalities and VAs, to symptomatic patients in whom SCD can be the first manifestation of the disease. An inappropriate adrenergic innervation may contribute to arrhythmogenesis in AC because it enhances propensity for VAs, particularly during sport and catecholamine release [41]. Later in the disease evolution, progressive deterioration of ventricular performance may result in right or biventricular heart failure [4].

### 3.1. Phases of the Disease

Different phases of the disease are recognized and include: (1) The *pre-histologic phase*, which encompasses the time period between birth and the beginning of the disease process; (2) the *preclinical phase,* during which initial histologic changes of the myocardium (myocyte death and fibro-fatty replacement) can be observed, although still clinically concealed; (3) the *pre-symptomatic phase*, when first electrical (ECG changes and premature ventricular beats) and then structural abnormalities (dilatation and dysfunction of the ventricles, either global or segmental), without relevant symptoms, become overt; (4) the *symptomatic phase*, which is characterized by symptoms, VAs and SCD, commonly occurring in adolescents with an overt disease phenotype [42,43]. Pre-participation athletic screening including an electrocardiogram, and cascade family screening after identification of probands, aim to diagnose patients in their pre-symptomatic (silent) phase and prevent fatal events, by sport disqualification, anti-arrhythmic drugs and ICD implantation in high-risk patients [44].

### 3.2. Age-Related Arrhythmic Manifestations

The ventricular electrical instability is the main determinant of the prognosis in AC, and life-threatening VAs can occur any time during the disease course. The risk of SCD reaches its peak between age of 21 and 35 years; several predictors of SCD have been identified and include a history of atrial fibrillation, syncope of probable cardiac origin, participation in strenuous exercise after the diagnosis, electrocardiographic T-wave inversions >V3, moderate to severe right or left ventricular dysfunction, hemodynamically tolerated sustained ventricular tachycardia (VT) and male gender [45,46]. 

Interesting age-related differences in arrhythmic manifestations have been observed and should be taken in mind while evaluating AC patients [44,47,48]. Cardiac arrest by ventricular fibrillation (VF) is mostly reported during the earlier phases of AC, whereas sustained VTs occur more commonly later in the disease course. Bhonsale et al demonstrated that AC patients experiencing VF and SCD were significantly younger (median age 23 years) than those presenting with sustained monomorphic VTs (median age 36 years) [47]. A subsequent study by the same group showed that in patients with late presentation (>50 years) sustained VT was the predominant arrhythmic event, in contrast to the young people, in whom VF was more common [48]. A pathobiological explanation of this particular age-related arrhythmic behavior may be due to the progressive nature of the disease, which modifies the myocardial lesions over time. While sustained monomorphic VTs are caused by re-entry circuits around stable fibrofatty myocardial scars, as a result of a healing process taking place in more advanced stages of the disease, VF may be the result of acute electrical instability, particularly in the context of myocarditis-mediated bouts of acute myocyte necrosis [49]. In fact, in the early stages of AC, probands and healthy gene mutation carriers (mostly *DSP*) may exhibit a particular susceptibility to suffer “hot phases” of chest pain and release of troponins, closely resembling clinically acute myocarditis with infarct-like presentation [50,51,52]. Virus-negative myocarditis is reported in a high proportion of histologically proven AC and myocardial inflammation has been documented in transgenic animal models with AC, as a response to myocyte necrosis [53,54]. Moreover, recent data proved the presence of autoimmunity in AC probands and affected members and it was associated with more severe forms of disease, frequent VAs and symptoms [55,56].

Nevertheless, experimental animal studies observed that SCD due to VF may occur even in the pre-histologic phase, before the development of fibrofatty myocardial lesions, because of a cross-talk of the mutant desmosomal proteins with the voltage-gated sodium channel complex and generation of arrhythmias with mechanisms similar to those described in Brugada syndrome [20,57,58]. It remains to be established, however, if the mechanisms leading to VF during the so-called “concealed disease” in humans are primarily electrical or caused by early, clinically silent, changes occurring at a histologic level. If on the one hand, mice with cardiac overexpression of mutant *DSG2* showed VAs only if aged ≥6 weeks and after histopathological demonstration of myocardial scar (*pre-clinical, histologic phase*) [19,54], on the other, mutations in *DSP* and *PKP2* were found in young SCD victims with negative autopsy, thus supporting the hypothesis of an arrhythmogenic mechanism acting at a molecular level and preceding the histologic phase [59]. 

### 3.3. Disease Phenotypes

#### 3.3.1. Classic Right Dominant Phenotype

The *classic RV phenotype*, which accounts for about 30% of AC diagnosis, is characterized by the isolated or predominant RV involvement and by the following clinical features:

1. ECG repolarization anomalies like T-wave inversion (TWI) in right precordial leads (V1-V4);
2. ECG depolarization anomalies like the epsilon wave or delayed terminal activation duration (TAD) of QRS;
3. VAs, such as frequent premature ventricular beats or VTs, having a left bundle branch block and superior axis morphology;
4. RV morpho-functional abnormalities (segmental RV wall motion abnormalities plus RV dilatation or dysfunction) on imaging tests such as contrast echocardiography, CMR or cine-ventriculography [5]. The ECG features typically precede the morpho-functional abnormalities, becoming more extensive with the progression of the disease. Interestingly, a higher RV dilatation and lower RV ejection fraction are associated with the extent of TWI toward left precordial and inferior leads, and prolonged TAD [60].

#### 3.3.2. Biventricular or Left Dominant Variants

The *biventricular* and the *left-dominant* variants differ from the classic ARVC because the pathological changes of ventricular myocardium do not remain confined to the RV, but involve in parallel or predominantly the LV. In a recent comprehensive autopsy study, LV involvement was observed in the overwhelming majority of AC-related SCD victims [61] and was the exclusive pathological finding in almost a fifth of cases. Given that a normal macroscopic appearance of the heart was found in 17% of SCD cases, an expert pathological evaluation, including histology, has a central role in the diagnosis in cases of unexplained SCD. Moreover, these findings give further evidence that LV variants of AC may evade clinical detection using current diagnostic tools [61].

**Figure 3:**
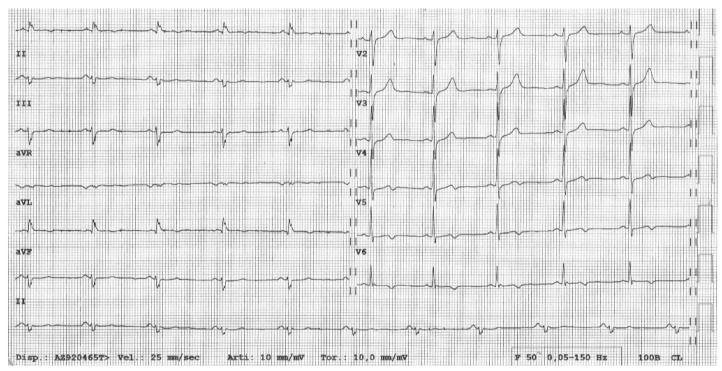
Electrocardiogram of patient with desmoplakin (*DSP*)-related left dominant arrhythmogenic cardiomyopathy. Basal electrocardiogram showing T-wave inversion in lateral leads and low QRS voltages (<0.5 mV) in limb leads.

Although specific diagnostic criteria for the non-classical variants of AC are lacking, recent studies addressed the distinctive features of AC-related LV phenotype, which can be here summarized:

1. ECG TWI in the (infero)lateral leads (II, III, avF, and/or V4-V6) [62,63];
2. Low QRS voltages (<0.5 mV) in limb leads (Figure 3) [32,60,62]
3. VAs with a right bundle branch block morphology, reflecting the origin from the LV [62,64];
4. Morpho-functional imaging features consistent with a “hypokinetic, non-dilated, and fibrotic LV”, namely a LV showing a mild systolic dysfunction, no (or mild) dilatation, and a great amount of subepicardial/mid-myocardial (non-ischaemic) LGE [29,30,33,62,63,64].

**Figure 4:**
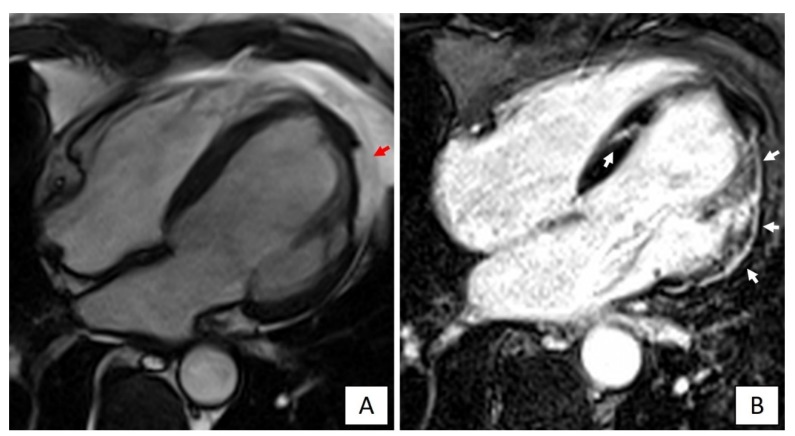
Cardiac magnetic resonance imaging of a patient with *DSP*-related left dominant arrhythmogenic cardiomyopathy. (**A**) End-diastolic frame of cine cardiac magnetic resonance sequence in long-axis four-chamber view showing fatty infiltration of the lateral wall of the left ventricle (red arrow). (**B**) Post-contrast image showing myocardial fibrosis in the form of extensive late gadolinium enhancement in the lateral wall and septum of the left ventricle (white arrows).

These characteristics substantiate the concept that the AC phenotype differs from that of DCM, being distinctively characterized by a large amount of LV myocardial fibrosis with prevalent subepicardial distribution, scarcely affecting the global LV systolic function, but acting as primary substrate for life-threatening VAs [63]. The recognition of the correct LV phenotype has significant implications for treatment, including indication for ICD that in AC patients may be considered even if the LV systolic function is not severely depressed [7]. With this regard, it must be emphasized that an imaging approach limited to a mere evaluation of the LV function, either global or regional, by echocardiography or cine-CMR appears insufficient to detect LV involvement. Conversely, LGE-CMR increases the diagnostic sensitivity because it allows identification of non-transmural LV scars. Since these scars commonly spare the subendocardial layers that mostly contribute to myocardial thickening, they may be undetectable by echocardiography (Figure 4) [63,64]. 

## 4. Impact of Sports Activity 

Sport is the most important environmental factor enhancing AC progression and worsening the disease arrhythmic substrate. The risk of SCD is known to be 5-fold higher in AC adolescent and young adults that practice competitive sports activity [65]. Ruwald et al. showed that participation in competitive sport is associated with an absolute risk of potentially lethal arrhythmic events of 61% at 40 years of age in AC patients [66]. In fact, if a genetically determined impairment of cell-to-cell adhesion is present, the mechanical stress generated during physical activity may promote myocyte death. Kirchhof et al. demonstrated that in heterozygous plakoglobin-deficient mice endurance training facilitated the development of RV abnormalities and VAs [67]. Endurance training in AC patients, especially high intensity exercise, has been associated with VAs, more severe RV dysfunction, LV dysfunction, and heart failure [68,69]. Conversely, the risk of VAs and mortality can be reduced by lowering exercise [42,66,69,70]. A dose-dependent relationship between exercise exposure and the disease penetrance was demonstrated in different categories of AC patients. Among genotype-positive relatives, clinical studies showed that competitive sports and high-intensity physical exercise increased age-related penetrance, risk of VAs and heart failure [41,71]. Thus, presymptomatic genetic testing may not only enable early diagnosis but also decrease the risk of developing AC through lifestyle changes. 

So, preparticipation screening and disqualification from competitive sports activity may prevent SCD by early identification of affected patients [72]. Accordingly, both European and American recommendations for sports eligibility in patients with heart diseases and the International consensus documents on AC treatment agree that restriction from competitive sports activity of AC patients should be considered a therapeutic measure aimed to reduce the risk of SCD [1,6,73,74]. 

## 5. Conclusions

In this review, we summarized several aspects of AC in relation to genetic basis, clinical course and disease phenotypes. In the recent years, there have been important advances in the understanding of genetic basis of AC, which contributed to further clarify the pathophysiology and better define the disease phenotype. Specific gene mutations have been associated with LV variants of AC, in some cases showing a particular propensity for VAs, and hence requiring an early aggressive anti-arrhythmic therapy. Natural history of AC recognizes different clinical phases, which should be taken in mind while treating these patients, for a better risk stratification and a more effective SCD prevention.
